# Cardiac Amyloidosis Mimicking Non-ST-Segment Myocardial Infarction: A Case Report

**DOI:** 10.7759/cureus.64097

**Published:** 2024-07-08

**Authors:** Argyroula Karampela, Nikos Adamidis, Sofia Adamidi, Sotirios Adamidis

**Affiliations:** 1 First Department of Internal Medicine, Athens Medical Group, Athens, GRC; 2 First Department of Internal Medicine, Sismanogleio Hospital, Athens, GRC; 3 Department of Internal Medicine, Charlton Memorial Hospital, Massachusetts, USA

**Keywords:** nstemi, electrocardiography, troponin, myocardial infiltration, cardiac amyloidosis

## Abstract

Cardiac amyloidosis is a rare but increasingly recognized condition characterized by the deposition of amyloid fibrils in cardiac tissue, leading to structural and functional heart impairment. This infiltrative cardiomyopathy often mimics more common cardiac conditions, posing significant diagnostic challenges. Particularly deceptive is its presentation as non-ST-segment elevation myocardial infarction (NSTEMI), where the clinical overlap necessitates considering amyloidosis in differential diagnoses. A 75-year-old male presented with muscle weakness, respiratory infection symptoms, and elevated cardiac enzymes. His history included a recent hospitalization for NSTEMI, with normal coronary angiography. Initial evaluations showed elevated troponin and CRP levels. A comprehensive cardiac assessment revealed a dilated ascending aorta, moderate systolic dysfunction (left ventricular ejection fraction (LV-EF), 47%), and asymmetrical interventricular septal thickening, suggesting hypertrophic cardiomyopathy or amyloidosis. The patient improved and was referred for further specialized care. Cardiac amyloidosis can mimic acute coronary syndrome (ACS), presenting with chest pain and elevated cardiac biomarkers. Differentiation is critical as amyloidosis involves myocardial infiltration by amyloid proteins, leading to restrictive cardiomyopathy. Advanced imaging techniques like cardiac MRI and nuclear scintigraphy are essential for accurate diagnosis and appropriate management, impacting therapeutic strategies and patient outcomes.

## Introduction

Cardiac amyloidosis is a rare but increasingly recognized condition characterized by the extracellular deposition of amyloid fibrils within the cardiac tissue, leading to structural and functional heart impairment. This infiltrative cardiomyopathy can present with a wide array of clinical manifestations, often mimicking more common cardiac conditions, thereby posing significant diagnostic challenges. Among these, the presentation of cardiac amyloidosis as an acute coronary syndrome (ACS), specifically non-ST-segment elevation myocardial infarction (NSTEMI), is particularly deceptive and warrants close clinical attention [1, 2].

Elevated cardiac biomarkers without persistent ST-segment elevation on the electrocardiogram (ECG) are commonly identified as NSTEMI, which is typically associated with partial obstruction of coronary arteries [3]. The clinical overlap between NSTEMI and cardiac amyloidosis arises because both conditions can present with similar symptoms, such as chest pain, dyspnea, and elevated cardiac troponin levels [4]. However, the underlying pathophysiology differs markedly, with amyloidosis involving myocardial infiltration by amyloid proteins, which can lead to restrictive cardiomyopathy, heart failure, and arrhythmias [5]. This similarity in clinical presentation, but a difference in etiology, underscores the importance of considering amyloidosis in the differential diagnosis of NSTEMI.

Immunoglobulin light chains (in light chain amyloidosis (AL)) or transthyretin (in transthyretin amyloidosis (ATTR)) most commonly compose amyloid fibrils in cardiac amyloidosis. The myocardial infiltration by these proteins leads to thickened ventricular walls, impaired diastolic function, and eventually systolic dysfunction as the disease progresses [6]. This pathology can obscure the clinical picture, making cardiac amyloidosis a master of disguise among cardiac diseases.

This case report aims to highlight the diagnostic complexity and clinical significance of cardiac amyloidosis presenting as NSTEMI. By examining a specific case, we emphasize the importance of including amyloidosis in the differential diagnosis for atypical or treatment-resistant cardiac cases. Through this discussion, we aim to enhance clinician awareness and promote more accurate diagnosis and management strategies for patients with this rare yet critical condition.

## Case presentation

A 75-year-old male patient was admitted to the Emergency Department presenting with reduced muscle strength in his lower right extremity, symptoms indicating lower respiratory tract infection, and elevated cardiac enzymes.

His medical history included a recent hospitalization at the University General Hospital of Patras for NSTEMI and respiratory infection. During his initial hospitalization, a coronary angiography revealed no critical stenosis. However, the patient exhibited symptoms of diplopia due to right ocular nerve paresis. A magnetic resonance imaging (MRI) of the brain conducted at that time showed a recent cerebral infarction in the mesencephalon extending to the left thalamus.

Admission laboratory tests at the Emergency Department revealed elevated troponin levels (3,168.8 pg/ml, normal range 24-30 pg/ml) and increased levels of C-reactive protein (CRP) (67.4 mg/L, normal range 0-5 mg/L), indicating ongoing inflammation and myocardial damage. The patient’s renal function tests showed a serum creatinine level of 1.2 mg/dL (normal range 0.6-1.2 mg/dL) and a blood urea nitrogen (BUN) level of 20 mg/dL (normal range 7-20 mg/dL). The patient's elevated cardiac biomarkers and clinical presentation prompted us to include sepsis-induced demand ischemia in the differential diagnoses, and appropriate evaluations were conducted to rule out this condition.

A chest X-ray was performed and showed no significant abnormalities. A computed tomography (CT) scan of the chest indicated bronchiolitis, with findings of inflammation in both lungs but no significant lymphadenopathy.

The patient was initially treated for a respiratory infection with piperacillin-tazobactam. Due to the isolation of *Staphylococcus epidermidis *in blood cultures, the patient also received vancomycin. The FilmArray for lower respiratory tract infection revealed parainfluenza virus 3.

Throughout his stay at our hospital, the patient underwent comprehensive cardiac and neurological evaluations. Doppler ultrasound of the carotid arteries revealed mild atherosclerotic changes without significant stenosis. The venous assessment of the lower extremities showed a history of saphenous vein stripping with significant incompetence detected via the Valsalva maneuver.

**Figure 1:**
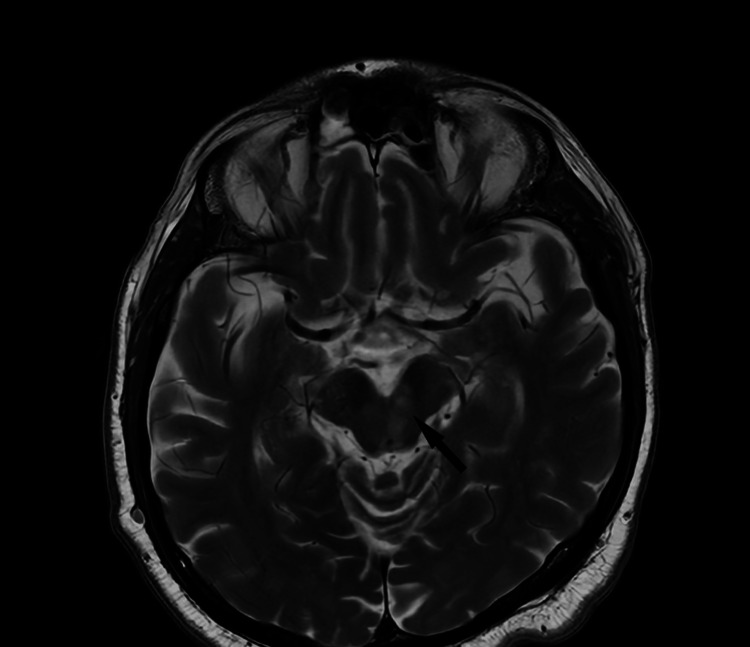
Magnetic resonance tomography of the brain T2-weighted axial scan of the brain: Arrow shows small ischemic lesion in the midbrain

Brain MRI and magnetic resonance angiography (MRA) were performed, revealing small ischemic lesions in the midbrain and the right parietal lobe, consistent with recent ischemic events (Figure 1). No atrial fibrillation (AF) was detected on admission.

**Figure 2:**
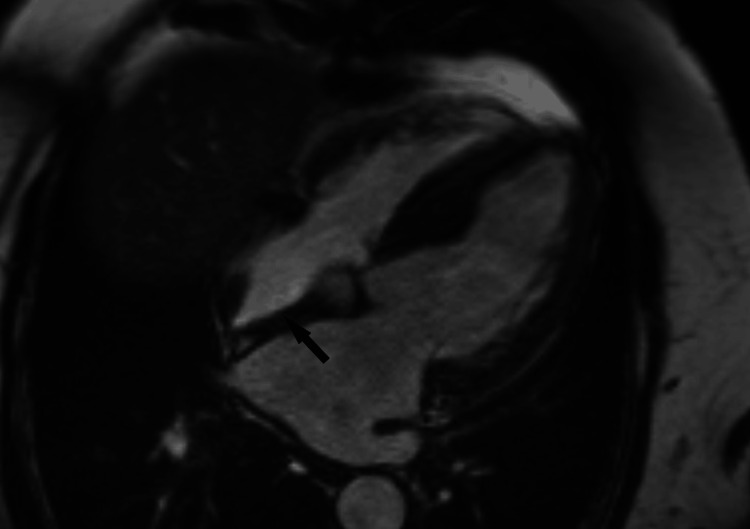
Cardiac magnetic resonance tomography T2-weighted images axial scan of the heart: Arrow shows hyperintense areas within the myocardium

**Figure 3:**
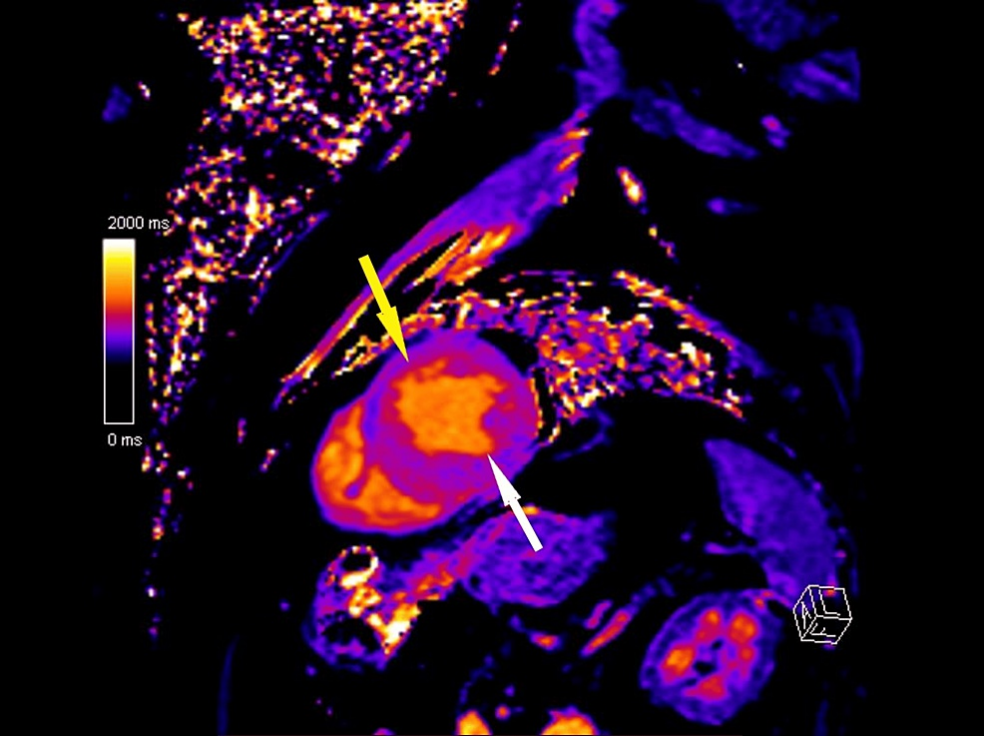
Cardiac magnetic resonance imaging (MRI) T1 map The bright orange area in the myocardium suggests prolonged T1 relaxation time (white arrow). The surrounding purple and blue areas represent normal myocardium with shorter T1 relaxation times (yellow arrow)

A cardiac MRI was also performed, revealing dilation of the ascending thoracic aorta and moderate systolic dysfunction with a left ventricular ejection fraction (LV-EF) of 47%. Significant asymmetrical thickening, particularly in the interventricular septum (IVS) (19 mm), suggested hypertrophic cardiomyopathy or an infiltrative process. MRI findings indicated widespread myocardial infiltration, raising the possibility of an infiltrative cardiomyopathy such as amyloidosis (Figures 2-3).

Upon discharge, the patient showed improvement in respiratory function and a slight decrease in cardiac enzyme levels. Final laboratory tests indicated stabilized blood parameters, with a troponin level of 2,082.3 pg/ml and CRP of 7.9 mg/L. The patient was referred to continue his care at a specialized cardiology center for further investigation and management of his cardiac condition.

At the diagnostic workup at the specialized cardiology center, the cardiac MRI showed late gadolinium enhancement consistent with amyloid deposition, and the fat pad biopsy was positive for amyloid with Congo red staining.

## Discussion

A review of the literature reveals several instances where cardiac amyloidosis has masqueraded as NSTEMI. For instance, Tew et al. reported a case of a 53-year-old man who presented with chest pain, ECG changes, and a slight rise in cardiac troponin I levels, initially suggesting acute coronary syndrome. However, investigations revealed left ventricular hypertrophy (LVH) and unusual patterns on cardiac MRI. Ultimately, primary AL amyloidosis with cardiac involvement was diagnosed, confirmed by serum-free kappa light chain levels and amyloid deposits in the bone marrow. The patient received chemotherapy and remained stable for over a year. This case underscores the importance of considering infiltrative cardiomyopathies in the differential diagnosis when traditional risk factors for coronary artery disease are absent [1].

Similarly, George et al. described a 75-year-old man presented with chest pain, initially diagnosed as NSTEMI ACS due to ECG changes and elevated cardiac markers. Despite treatment, recurrent symptoms and further investigations revealed mild coronary artery disease, LV dysfunction, and ultimately, cardiac amyloidosis diagnosed through various imaging techniques and histological confirmation. The patient was treated with heart failure medications and chemotherapy but continued to experience recurrent symptoms [2].

In another notable case, Nguyen et al. detailed A 72-year-old man presented with chest discomfort and dyspnea, initially suspected to be acute coronary syndrome (ACS). Despite typical symptoms and elevated cardiac troponin levels, coronary angiography revealed nonobstructive coronary arteries. Further clinical evaluation, including the presence of macroglossia and periorbital purpura, and laboratory tests indicating elevated serum-free light chain levels, confirmed a diagnosis of AL amyloidosis through histological analysis of an abdominal skin biopsy [7].

Cardiac amyloidosis is a condition where abnormal protein deposits, known as amyloid, accumulate in the heart tissue, leading to various cardiac issues such as heart failure, arrhythmias, and cardiomyopathy. The condition can present with thromboembolic events, including strokes, due to the increased risk of intracardiac thrombosis [8]. Suleiman et al. reported a 72-year-old man presented with right-sided weakness, aphasia, and gaze palsy, initially diagnosed as an ischemic stroke due to AF. Further investigation revealed cardiac amyloidosis as the underlying cause. Despite being on anticoagulation therapy, he experienced recurrent strokes. Diagnostic tests, including echocardiography, cardiac MRI, and a positive fat pad biopsy, confirmed cardiac amyloidosis. The patient was treated for heart failure and received a cardiac resynchronization therapy (CRT) defibrillator [8].

Diagnosing cardiac amyloidosis can be particularly challenging due to its varied clinical presentation and overlap with other cardiac conditions. NSTEMI, characterized by partial obstruction of coronary arteries, elevated cardiac biomarkers, and absence of persistent ST-segment elevation on ECG, shares symptoms with cardiac amyloidosis such as chest pain and dyspnea [9]. However, the underlying pathophysiology in amyloidosis involves myocardial infiltration by amyloid fibrils, leading to restrictive cardiomyopathy and heart failure [6].

One major challenge is the nonspecific nature of the symptoms and signs. Traditional diagnostic approaches for ACS, such as ECG and serum biomarkers, may not differentiate between ischemic and infiltrative processes. Elevated troponin levels, a hallmark of myocardial injury, are commonly observed in both NSTEMI and cardiac amyloidosis. However, in amyloidosis, the troponin elevation is due to direct myocardial damage from amyloid deposits rather than ischemia [10].

Imaging studies play a crucial role in the diagnostic workup. Echocardiography can reveal characteristic features of amyloidosis, such as increased ventricular wall thickness and a "sparkling" myocardial appearance, although these findings are not specific [11]. Cardiac MRI is more definitive, providing detailed tissue characterization and identifying amyloid infiltration through late gadolinium enhancement patterns [12]. In certain cases, nuclear imaging with technetium-labeled bone tracers can differentiate ATTR amyloidosis from AL amyloidosis, aiding in appropriate management [13].

The misdiagnosis of cardiac amyloidosis as NSTEMI can have significant clinical implications. Patients with amyloidosis often require different therapeutic approaches compared to those with ischemic heart disease. For instance, while antiplatelet therapy and revascularization are mainstays in treating NSTEMI, these interventions are not beneficial in amyloidosis and may even pose risks, such as bleeding complications [14]. Furthermore, the prognosis and treatment options differ markedly between the two conditions. AL amyloidosis often necessitates chemotherapy and hematopoietic stem cell transplantation to target the underlying plasma cell dyscrasia [15]. In contrast, ATTR amyloidosis may be treated with novel pharmacological agents like tafamidis, which stabilize the transthyretin protein and slow disease progression [16].

Early and accurate diagnosis is therefore essential to guide appropriate therapy and improve patient outcomes. Nuclear imaging with technetium-99m pyrophosphate (Tc-99m PYP) scintigraphy has proven particularly useful in distinguishing ATTR amyloidosis from AL amyloidosis [17]. High uptake of Tc-99m PYP is characteristic of ATTR amyloidosis, whereas AL amyloidosis typically shows low or absent uptake. This differentiation is critical as it directly impacts treatment strategies and prognosis.

The clinical outcomes in these cases varied significantly based on the underlying type of amyloidosis and the timeliness of diagnosis. Patients with AL amyloidosis who received early chemotherapy and stem cell transplantation showed improved survival rates compared to those with delayed diagnosis [18]. In contrast, patients with ATTR amyloidosis benefited from novel pharmacotherapies like tafamidis, which have been shown to reduce mortality and hospitalization rates in clinical trials [19].

## Conclusions

Cardiac amyloidosis mimicking NSTEMI represents a diagnostic and therapeutic challenge, requiring a high index of suspicion and a multidisciplinary approach. The reviewed literature underscores the importance of considering amyloidosis in patients with atypical cardiac presentations and persistent symptoms despite standard treatment for ACS. Advanced imaging techniques and specific biomarkers are essential for accurate diagnosis, guiding appropriate and timely management. Specifically, cardiac MRI with late gadolinium enhancement is highly useful for identifying amyloid deposits, while Tc-99m PYP scintigraphy helps distinguish between ATTR and AL amyloidosis. Biomarkers such as serum-free light chains and elevated troponin levels provide additional diagnostic clarity. Early recognition and targeted therapy significantly impact patient outcomes, emphasizing the need for increased awareness and continued research in this field. By integrating clinical, imaging, and histological data, clinicians can better differentiate cardiac amyloidosis from other cardiac conditions, ultimately improving prognosis and quality of life for affected patients.
